# Controlling the Outcome of the Toll-Like Receptor Signaling Pathways

**DOI:** 10.1371/journal.pone.0031341

**Published:** 2012-02-20

**Authors:** Guilhem Richard, Calin Belta, A. Agung Julius, Salomon Amar

**Affiliations:** 1 Program in Bioinformatics, Boston University, Boston, Massachusetts, United States of America; 2 Department of Electrical, Computer and Systems Engineering, Rensselaer Polytechnic Institute, Troy, New York, United States of America; 3 Center for Anti-Inflammatory Therapeutics, Goldman School of Dental Medicine, Boston University, Boston, Massachusetts, United States of America; Indian Institute of Science, India

## Abstract

The Toll-Like Receptors (TLRs) are proteins involved in the immune system that increase cytokine levels when triggered. While cytokines coordinate the response to infection, they appear to be detrimental to the host when reaching too high levels. Several studies have shown that the deletion of specific TLRs was beneficial for the host, as cytokine levels were decreased consequently. It is not clear, however, how targeting other components of the TLR pathways can improve the responses to infections. We applied the concept of *Minimal Cut Sets* (MCS) to the *ihs*TLR v1.0 model of the TLR pathways to determine sets of reactions whose knockouts disrupt these pathways. We decomposed the TLR network into 34 modules and determined signatures for each MCS, *i.e.* the list of targeted modules. We uncovered 2,669 MCS organized in 68 signatures. Very few MCS targeted directly the TLRs, indicating that they may not be efficient targets for controlling these pathways. We mapped the species of the TLR network to genes in human and mouse, and determined more than 10,000 *Essential Gene Sets* (EGS). Each EGS provides genes whose deletion suppresses the network's outputs.

## Introduction

Signal transduction pathways, such as the Toll-Like Receptors (TLR) signaling pathways, are an essential component of the innate and acquired immune response [1]. The TLRs are highly conserved membrane receptors that recognize specific molecules of bacterial and viral origin. When triggered, the TLRs recruit adaptor molecules such as MyD88 or TICAM [2], [3], and initiate signaling cascades leading to the activation of transcription factors (TFs). Different stimuli activate specific sets of TFs, which regulate the necessary response of the cell.

Many studies have established the significant role TLRs play within the immune system: detection of pathogenic particles [3], signal transduction [4], and activation of TFs [5]. For some infections, recent works have shown that the TLRs provoke deleterious side effects to the host by increasing cytokine concentrations. They have established that mice deficient in one of the TLRs had a better response to infection: TLR4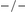 for *C. rodentium* [6], TLR2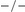 for *P. gingivalis* [7], and TLR3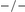 for *Phlebovirus* [8]. Targeting a TLR enables to decrease cytokine concentrations leading to an increased survival rate. These results demonstrate that regulating cytokine production may be a good strategy to improve responses to infections. One has to keep in mind that the deletion of a TLR has an overall negative effect on the host on the long term. Polymorphisms in the TLRs significantly increase the susceptibility to opportunistic infections [9], emphasizing the key role the TLRs play in the immune system. It is not clear, however, how targeting other components of the TLR pathways can improve the responses to infections.

The *ihs*TLR v1.0 model is a stoichiometric representation of the human TLR signaling pathways that follows six outputs: AP-1, CREB, IRF3, IRF7, Reactive Oxygen Species (ROS), and NF-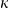
B (Table 1) [10]. These compounds play a major role in the response to infection. AP-1 is involved in proliferation and differentiation, and activates both pro- and anti-apoptotic responses [11]. CREB responds to growth factor signals and regulates cell survival and proliferation [12]. IRF3 and IRF7 are activated during viral infections and are critical for the activation of Type I IFN [13]. NF-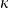
B plays a role in both innate and adaptive immune responses by regulating B- and T-cells development, and is involved in the inflammatory response [14]. ROS, which is the only output not being a TF, are highly reactive chemicals containing oxygen. ROS regulate signal transduction pathways at small dose, provoke oxidative damage at high dose, and are actively involved in wound healing processes [15].

**Table 1:** Output reactions of the *ihs*TLR v1.0 model.

Output name	Description
AP-1	Binding of the Jun/Fos dimer to the AP-1 site
AP-1(2)	Binding of the Jun/Jun dimer to the AP-1 site
CREB	Binding of CREB to the CRE site
IRF3	Binding of phosphorylated IRF3 to the ISRE site
IRF7	Binding of phosphorylated IRF7 to the ISRE site
NF- 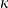 B	Dissociation of the NF- 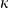 B/I 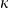 B 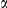 complex
NF- 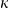 B(2)	Dissociation of the NF- 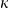 B/I 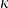 B 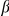 complex
ROS	Formation of the NADPH oxidase complex, the p47 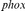 subunit being phosphorylated three times
ROS(2)	Formation of the NADPH oxidase complex, the p47 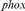 subunit being phosphorylated eight times

The stoichiometric reaction format has been used extensively to represent metabolic [16], [17] and signaling networks [18]–[20]. Alternatively, signal transduction pathways have been modeled by Petri nets [21] and Boolean logic [22]. The stoichiometric reaction format has led to the development of various methods, such as Extreme Pathway (EP) analysis [23], Flux Balance Analysis (FBA) [24], and Minimal Cut Sets (MCS) [25]. An MCS for an objective reaction is a minimal set of reactions whose knockout disables that function. This notion is helpful for studying robustness and epistasis relationships in complex networks. FBA can easily generate MCS by testing all knockout combinations in a network, however, this approach becomes computationally challenging for large-scale networks. EP analysis can bypass this difficulty and has successfully been applied to the *E. coli* and human metabolic models [25]–[27].

In this study, we generated MCS for the *ihs*TLR v1.0 model. We encountered 2,669 MCS showing that each output uses different components of the TLR network. We partitioned the model in *modules*, which group reactions having similar functions. We determined which modules the MCS targeted, allowing to identify epistatic relations between components of the network. It appeared from our analysis that the TLRs were not the primary targets of the MCS, implying the TLR pathways are better manipulated by disabling targets downstream of the TLRs. We extended the MCS to the notion of *Essential Gene Sets* (EGS). EGS are sets of genes whose deletion ensures the knockout of an output. We assessed the impact of these deletions over the network, in term of species, reactions, and modules perturbed. We generated more than 10,000 EGS for both human and mouse. The EGS provide valuable information when designing knockout experiments by identifying gene deletions that have minimal impact on the network. They also identify essential genes that have a key role in the activation of a particular output of the TLR pathways.

## Results

### 2,669 minimal cut sets produced 68 different signatures

The *ihs*TLR v1.0 model comprises 781 species involved in 963 reactions. We extended the model to 1,956 reactions to account for irreversibility and sink reactions. The over-approximation flux cone 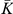 of the model yielded 1,598,509 EPs. We computed hitting sets for each output reaction and reduced them to minimality. We obtained a total of 2,669 MCS for all outputs (Table 2) (see Materials and Methods). The number of MCS greatly varies across outputs: it ranges from 6 for NF-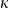
B(2) to 1,042 for IRF7.

**Table 2:** Basic statistics about the MCS.

Output	MCS	Cardinality	Signatures	Module hit
AP-1	189	1–7	10	7
AP-1(2)	185	1–7	9	7
CREB	510	1–8	18	9
IRF3	22	1–3	6	5
IRF7	1,042	1–8	18	8
NF- 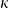 B	351	1–5	10	5
NF- 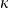 B(2)	6	1–3	1	1
ROS	138	1–6	21	13
ROS(2)	226	1–5	20	12
Total	2,669	1–8	68	24

The cardinality of an MCS is defined as the number of reactions present in the MCS. Its signature corresponds to the list of modules (cf. Table 3) that are targeted by the MCS. The table lists the number of distinct signatures obtained for each output reaction, as well as the total number of targeted modules.

**Figure 1:**
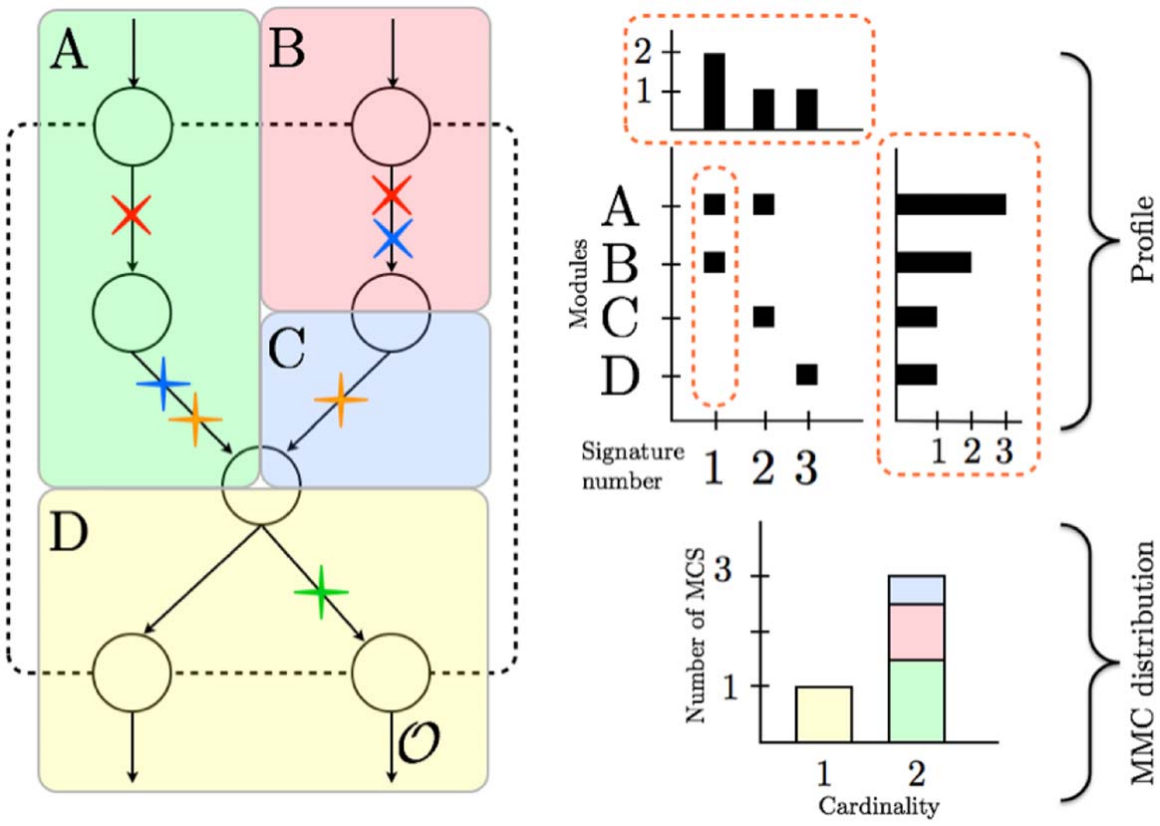
Schematic representation of a signaling network. The left part of the figure shows a toy model of a signaling network. Nodes and edges represent species and reactions, respectively. The network contains two inputs (top incoming arrows) and two outputs (bottom outgoing arrows). The set of reactions is partitioned in four modules (A, B, C, and D). Each crossed reaction belongs to an MCS for output 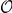 (only 4 MCS are shown for simplicity). Each color denotes a different MCS. Targeting simultaneously the two red-crossed reactions disables 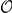. This MCS describes an epistatic relation between modules A and B. The upper-right diagram represents the profile of output 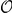, *i.e.* the collection of distinct signatures obtained from the MCS. The profile displays distributions showing the frequency of each signature (top) and how many times each module is hit by an MCS (right). In our example, the first signature is obtained twice (red and blue MCS). Module A is hit three times: twice by the first signature and once by the second one. The lower-right diagram represents the MCS Module Cardinality (MMC) distribution of 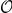, which shows the number of MCS targeting 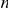 module(s) simultaneously (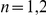). The targeted modules are color coded in relative proportion. Among all the MCS of cardinality 2, half of the targeted reactions belong to module A.

We grouped the reactions from the *ihs*TLR v1.0 into distinct *modules*. Reactions in the same module generally have the same function or lead to the same end product. These functions usually consist in activating a specific protein or in transmitting a stimulus to another part of the network. The modules determine a partition of the set of all reactions (see Fig. 1 for a module partition of a toy network). We derived 34 modules from the map of the TLR network (Fig. S1 by Li *et al.* [10]). This map clusters biologically related reactions that share substrates or products. Tables 3 and S1 provide the list and a description of the modules hit by MCS. Ten of the 34 modules are never targeted by any MCS: *Akt*, *Caspase 9*, *Cytoplasmic MAPK substrates*, *FADD*, *IL-1*, *IL-1 cascade*, *IKK*, *NOD*, *PKR*, and *SIGIRR*.

**Table 3:** Modules of the TLR network.

Module	Function
*AP-1*	Binding of AP-1 complex to AP-1 site
*Btk*	Activation of Btk
*Calcium dependent cascade*	Activation of calcium/calmodulin-dependent protein kinase
*Common metabolites*	Transport of metabolites into the cytoplasm
*CREB*	Binding of CREB to CRE site
*Cytoplasm-Nucleus transport*	Transport of various species into the nucleus
*Early endosome*	Degradation and recycling of receptors
*GSK3* 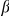	Activation of GSK3 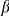
*IFN genes*	Binding of IFN with the ISRE site
*KSR1*	Activation of the MAPK pathway
*Ligands*	Import of ligands in the system
*Lipids*	Lipids phosphorylation
*MAPK*	Transmission of stimuli
*MAP3K7*	Transmission of stimuli
*MyD88*	Transmission of stimuli from the TLRs
*NF-* 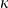 *B*	Dissociation of NF- 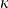 B from I 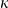 B
*NF-* 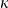 *B phosphorylation*	Activation of NF- 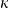 B
*PDK1*	Activation of PDK1
*PKC* 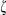	Activation of PKC 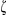
*Rho GTPases*	Activation of GTPases
*ROS production*	Formation of the NADPH oxidase complex
*Thioredoxin*	Oxidation and reduction of thioredoxin
*TICAM*	Transmission of stimuli from the TLRs and activation of IRF3
*TLR*	Binding of ligands with the TLRs

Function of the modules involved in the TLR network [10]. Only modules hit by MCS are shown. A short description of these modules is available in Table S1.

Three hypotheses are made. First, reactions in these modules may not be included in any EP, rendering them “unusable” in a cut set. The analysis of the partial flux cone shows, however, that the reactions in these modules are present in at least 1 EP. Second, these modules may not be essential for the activation of the outputs. Third, too many reactions from these modules may need to be targeted simultaneously to observe an effect. This last hypothesis reflects a limitation of the algorithm, which did not detect MCS of more than 8 reactions in our study.

**Figure 2:**
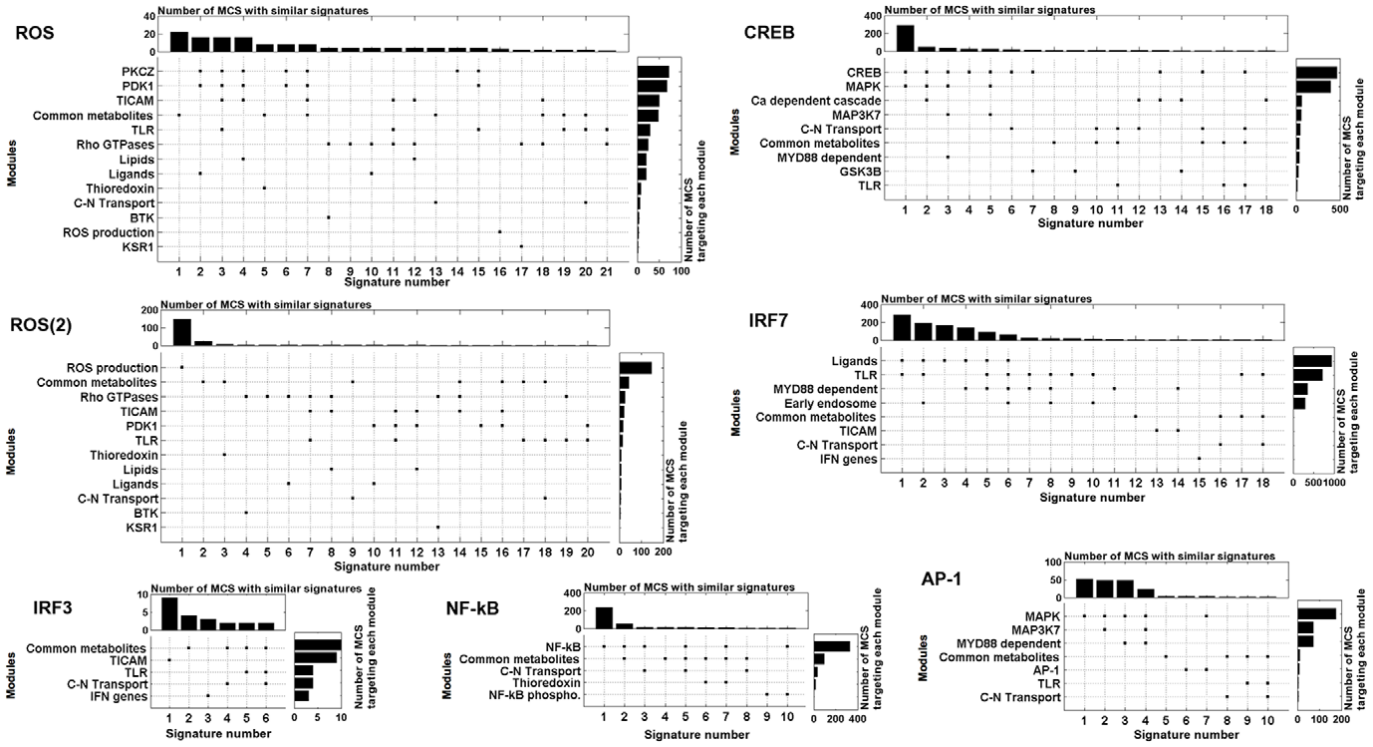
Profiles obtained for seven of the output reactions: AP-1, CREB, IRF3, IRF7, NF-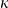
B, ROS, and ROS(2). Each vertical line represents a distinct signature, *i.e.* the list of modules hit by an MCS. The profiles also display distributions showing the number of times each signature is encountered (top) and how many times each module is hit by an MCS (right). These distributions emphasize the most frequent signatures and the most targeted modules, respectively (*cf.* Fig. 1). Profiles of AP-1(2) and NF-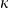
B(2) are omitted. The AP-1(2) profile is identical to the AP-1 one, minus the 7-th signature. The NF-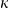
B(2) profile is composed of a single signature targeting the *NF-* 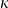 *B* module.

The mapping of reactions to modules enabled to determine the *signature* of each MCS (see Fig. 1 for a graphical illustration of this concepts in a small network). The signature of an MCS is the list of modules it targets, *i.e.* the modules containing reactions hit by the MCS. We obtained one signature per MCS. We were able to reduce the 2,669 MCS to 68 distinct signatures indicating that most of the MCS target the same combinations of modules. We define as the *profile* of an output the set of distinct signatures obtained for that output. We generated profiles for each output (Fig. 2). In addition to presenting the signatures, the profiles display distributions showing the number of times each signature is encountered (top) and how many times each module is hit by an MCS (right). These distributions emphasize the most frequent signatures and the modules that are targeted the most, respectively (*cf.* Fig. 1). The AP-1(2) and NF-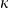
B(2) profiles are not shown in Figure 2. The AP-1(2) profile is identical to the AP-1 one, minus the 7-th signature. The NF-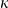
B(2) profile is composed of only one signature targeting the *NF-* 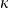 *B* module.

**Figure 3:**
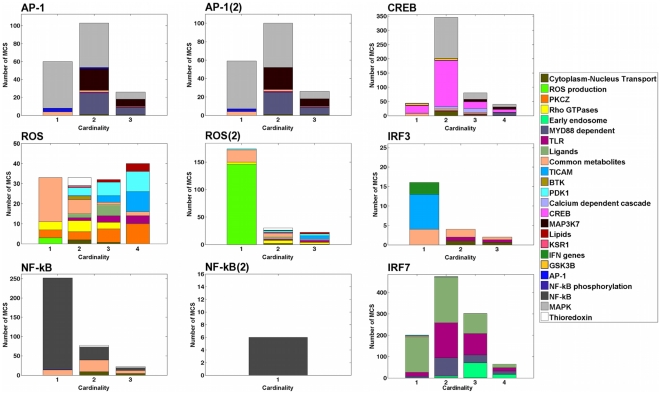
Distributions of the MCS Module Cardinality (MMC). Each reaction in a MCS belongs to a module and some of these reactions may belong to the same module. Hence, an MCS may target the same module through different reactions. The MMC represents the number of distinct modules an MCS hits. The bars in each plot represent the number of MCS targeting simultaneously 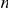 modules (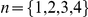). No MCS targets more than 4 modules at a time. The colors describe which modules are targeted, as well as their relative importance. The distributions enable to immediately identify modules that are targeted alone (*e.g. Common metabolites*, *ROS production*, *TICAM*) and the ones that are targeted with other modules (*e.g. PDK1* for ROS, *Early endosome* for IFR7, or *MyD88* for AP-1 and AP-1(2)). For 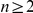, the distributions do no show which combinations of modules are hit together (see Fig. 2).

Each reaction in an MCS belongs to a module and some of these reactions may belong to the same module. Hence, an MCS may target the same module through different reactions. We define the *MCS Module Cardinality* (MMC) as the number of modules an MCS hits. Figure 3 shows the MMC distribution for the outputs. Each distribution describes how many MCS target 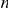 modules simultaneously for a given output (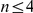 for all MCS). In addition, the colors describe which modules are targeted, as well as their relative importance. The distributions enable to immediately identify modules that are targeted alone (*e.g. Common metabolites*, *ROS production*, *TICAM*) and the ones that are targeted with other modules (*e.g. PDK1* for ROS, *Early endosome* for IFR7, or *MyD88* for AP-1 and AP-1(2)). Interestingly, the cardinality of the MCS does not seem to be a good indicator of the cardinality of the signature (*i.e.* the number of modules targeted simultaneously). Particularly for the CREB, ROS(2), NF-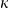
B, and IRF7 outputs, many MCS of high cardinality hit few modules at a time (Fig. S1). This observation seems to indicate that modules are fairly robust to reaction deletions.

### More than 10,000 Essential Gene Sets generated for human and mouse

**Figure 4:**
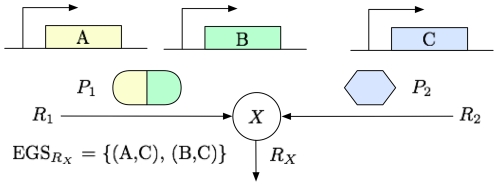
Identification of Essential Gene Sets. Species 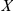 is produced by two distinct reactions: 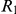 and 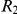. These reactions require proteins 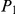 and 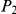 to be expressed, respectively. The proteins can be used as enzymes or substrates. 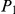 is a dimer coded by genes A and B, while 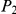 is coded by gene C. Species 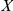 is no longer produced if both 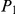 and 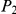 are absent. Hence, the deletion of genes A and C, or B and C renders reaction 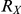 non-producible.

We define an *Essential Gene Set* (EGS) as a set of genes whose deletion renders a predefined output non-producible. EGS can be seen as the gene version of MCS. Instead of providing reactions, EGS procure the list of genes whose knockout stops the production of an objective, which is a valuable information when designing knockout experiments. We can clearly disable genes in an EGS (*e.g.* with siRNA), and the corresponding objective reaction, unlike reactions in an MCS (Fig. 4). EGS also summarize MCS since a common gene may be able to disable multiple reactions. Gene knockouts are more optimal than reaction knockouts. Finally, EGS identify essential genes that have a key role in the activation of a particular output of the TLR model. Even though genes have much slower dynamics than reactions in the TLR model, we do not perform any dynamical analysis of these pathways with the EGS. Transient behaviors of the network do not affect the producibility of the outputs.

The *ihs*TLR v1.0 model is based on human pathways. The TLRs and their pathways are however highly conserved across species and especially among vertebrates [28]. Since human and mouse share 90% of their genome [29], we generated Gene-Protein-Reaction (GPR) associations for the two organisms. Moreover, most data translation to human comes from mouse. We recognize that there are some significant differences between the human and mouse TLR pathways, notably in the TLR10 pathway [30]. This particular pathway was however not involved in the MCS we encountered, therefore limiting the inconsistencies in the mouse results. We were able to assign 341 and 335 genes to 570 and 567 species of the model in human and mouse, respectively (Dataset S1). The discrepancy in the number of relationships and genes is explained by different numbers of isoenzymes or subunits in both species. We used the MCS to generate initially 42 and 44 EGS for human and mouse, respectively (see Materials and Methods). The number of EGS is similar since all the proteins of the network have homologs in both organisms. We validated these EGS by searching the literature. In both species, 16 EGS involved genes directly coding for the outputs and required no further validation (*i.e.* “evident” EGS). We were also able to confirm a total of 12 EGS through literature searches (Tables 4 and S2). In summary, we were able to find evidence to validate 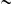
66% of all EGS.

**Table 4:** EGS validation.

Genes in EGS	Outputs targeted	Reference to validation experiments
MAPK8	AP1, AP1(2)	Dérijard *et al.* (1994) **JNK1: A protein kinase stimulated by UV light and Ha-Ras that binds and phosphorylates the c-Jun activation domain**, Cell 76: 1025–1037
TBK1, IKK 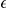	IRF3, IRF7	*Sharma *et al.* (2003) **Triggering the interferon antiviral response through an IKK-related pathway**, Science 300: 1148–51
TRAF6	IRF7	*Kawai *et al.* (2004) **Interferon-** 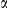 ** induction through Toll-like receptors involves a direct interaction of IRF7 with MyD88 and TRAF6**, Nat Immunol 5:1061–8
MyD88	IRF7	*Kawai *et al.* (2004) **Interferon-** 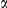 ** induction through Toll-like receptors involves a direct interaction of IRF7 with MyD88 and TRAF6**, Nat Immunol 5:1061–8
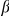 -TrCP	NF- 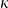 B(2)	Wu *et al.* (1999) 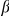 **-TrCP mediates the signal-induced ubiquitination of I** 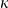 **B** 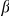 , J Biol Chem 274: 29591–4
Vav1	ROS, ROS(2)	Kim *et al.* (2003) **The hemopoietic Rho/Rac guanine nucleotide exchange factor Vav1 regulates N-formyl-methionyl-leucyl-phenylalanine-activated neutrophil functions**, J Immunol 171: 4425–30

Experimental validations of the predictions are found in the cited publications. A complete list of the EGS is shown in Table S2.

*These articles were also used for the reconstruction of the ihsTLR v1.0 model [10].

Only AP-1(2) failed to produce EGS targeting specifically this output. This is easily explained as disabling AP-1(2) requires to knock out the Jun protein. This protein is however essential for the formation of the Jun/Fos complex (*i.e.* AP-1, cf. Table 1). Hence, EGS found for AP-1(2) hit AP-1 as well. Since the ROS/ROS(2) and NF-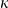
B/NF-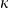
B(2) outputs are also closely related, many of the EGS for one of the outputs disable the other one.

Genes in the EGS are part of the MyD88-dependent pathway (*e.g.* MyD88, TRAF6, TLR7, TLR8, TLR9), MyD88-independent pathway (*e.g.* TICAM1, TBK1, IKK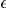), and the MAPK pathways (*e.g.* MAPK8, MAP2K4, MAP2K7). The initial EGS we computed contain in general a small number of genes (see Table S2). Disregarding the evident EGS, most of the remaining EGS contain no more than 3 genes (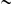
80%). It is experimentally difficult to knock out more than two genes at a time. Even with this limitation, 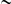
68% of the non-evident EGS remain experimentally usable.

We used the 42 and 44 initial EGS to generate a second set of EGS capable of disabling any combination of outputs. Combining the initial EGS gave us a total of 10,377 and 11,577 gene sets targeting any desired set of outputs in human and mouse, respectively. In addition, we computed the species, reaction, and module impacts of each EGS, *i.e.* the number of species, reactions, and modules disabled after deletion of the EGS. These values estimate the global impact of the EGS. We enumerated the 801 EGS that target the power set of outputs with minimal impact in both species (Dataset S2). The minimal EGS target a specific set of outputs while affecting a minimum number of species, reactions, and modules.

## Discussion

The TLR network is a complex set of signaling reactions which upon triggering leads to the activation of various TFs and release of cytokines. These cytokines, which coordinate the response to infection, also provoke collateral damage when reaching too high levels. Several studies have shown that the knockout of particular TLRs were beneficial for hosts undergoing specific infections, as it reduces cytokine concentrations [6]–[8]. It is not clear, however, how targeting other components of the TLR pathways can improve the responses to infections.

We studied the *ihs*TLR v1.0 model [10] and generated 2,669 MCS for all outputs of the network. The MCS were organized into 68 distinct signatures, revealing epistatic relationships in the network (Figs. 2 and 3). Epistasis was not examined at the reaction level, but rather at the module level. We focused our attention on the sub-systems of the network whose joint perturbation disabled an output. Epistatic relationships reveal the major functions needed to ensure the production of the outputs. For example, IRF3 production is shut down by perturbing the transmission of the stimuli from the TLRs via the TICAM adaptor molecules, while disabling the activation of both PDK1 and PKC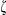 stops ROS production. Figure 3 shows how the network is being used for each output. Based on the output, different sets of modules are targeted. This difference in module composition suggests that each output uses different pathways of the network.

Since the TLRs are the inputs of the TLR network, one would expect that they control the outputs. Among the 2,669 MCS, only 183 targeted directly one of the TLRs. Even though the TLRs are critical in the response to infection, it appears that disabling them is inefficient to accurately control the network's outcome. Searching for targets downstream of the TLRs appears to be a better strategy to efficiently shut down a desired pathway. This observation may seem contradictory to previous works showing that the deletion of the TLRs perturbed reactions within these pathways. These studies, however, analyzed the TLR pathways for specific infections. Here, the MCS ensure the suppression of an objective regardless of the set of triggered TLRs, which makes MCS non-specific to a particular infection. The low frequency of the TLRs appearing in the MCS may also be due to redundancies in the TLR pathways, already highlighted in [10]. In their study, Li *et al.* showed that some outputs of the TLR network (*i.e.* NF-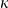
B, CREB, AP-1, and ROS) are activated by at least 11 different receptors. Hence, knocking out one TLR will not affect these outputs since the remaining receptors can potentially activate them. One would need to disable all the TLRs to ensure that the outputs can no longer be activated. This extreme solution has also the biological disadvantage of disabling all TLR pathways, making the host highly susceptible to infection. It is difficult to establish clear consequences of TLR knockouts on the network. We can only perform this analysis for IRF7. The MCS confirmed that the knockout of TLR7, TLR8, and TLR9 shuts down IRF7 activation, which disables the transcription of Type I IFN genes [13], and interferes with the differentiation of monocytes to macrophages [31].

Gene deletions can be performed to ensure the knockout of the outputs of the TLR network. Adequate combinations of genes, which we provide with the EGS, enable to target a specific set of outputs. Using the MCS, we generated 10,377 and 11,577 EGS for human and mouse, respectively. Since we considered a human TLR network, discrepancies may arise in the mouse EGS. This limiting factor is attenuated as the MCS did not involve any human specific pathway. The EGS identify essential genes for the activation of the network's outputs (Table S2). For example, TBK1 and IKK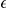 seem to be essential for the activation of IRF3 and IRF7, while PDK1 and PKC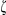 appear to be key in the production of ROS.

To the best of our knowledge, this study is the first attempt to use MCS for the study of signaling networks in the form of a stoichiometric model. MCS have initially been developed for the study of metabolic networks. Their application to the *E. coli* and *H. sapiens* metabolic networks have given interesting results [25]–[27]. Even though MCS were not intended for signaling networks, their usage on such networks gives promising results. Indeed, our validation of the EGS (derived from the MCS) showed that about 80% of them concord with biological observations. This validation process indicates that the usage of MCS on the *ihs*TLR v1.0 model provides results that are biologically relevant.

Li *et al.* defined *DIOS pathways* based on a pairing of inputs and outputs [10]. In our study, MCS and EGS are output-specific. However, we can compare the two approaches based on which output is controlled by the DIOS pathways. By using FBA, Li *et al.* only provided single reactions controlling a pathway. We analyzed EPs to determine sets of reactions, or MCS, disabling a particular output and to study epistatic relationships between components of the system. We obtained MCS containing up to 8 reactions. Obtaining similar results with FBA would be computationally very expensive for large-scale networks, such as the *ihs*TLR v1.0 model. In addition to control reactions we also provided (sets of) control genes, *i.e.* the EGS. In addition, Li *et al.* focused on single DIOS pathways, while we also describe gene sets capable of disabling multiple outputs.

We observed similarities with the essential reactions as reported in Table S6 by Li *et al.* [10]. Our EGS list that the evident knockout of IRF3 is capable of disabling the production of this output. IRF3 is also involved in all the essential reactions of the *IRF3* DIOS pathway. A similar result is observed with IRF7. Four out of the 5 essential reactions in the *ROS production* DIOS pathway are listed in our MCS. Our EGS list that the knockout of IKK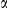, IKK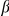, and IKK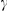 disables the NF-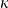
B output. These genes are the subunits of the IKK complex, which is involved in essential reactions of the *RIP1*, *NOD1*, *NOD2*, and *RIP2/TRIP6/TRAF2* DIOS pathways (two reactions in each pathway). These 4 DIOS pathways each control NF-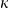
B [10]. Interestingly, we obtain similar results even after increasing the number of reactions in the model.

As suggested by Li *et al.*, metabolites seem to play a crucial role in signal transduction [10]. Only targeting the *Common metabolite* module, which perturbs the normal exchange of metabolites between the extracellular and cytoplasmic compartments, can disable most of the outputs. Interestingly, no other module has such effect, highlighting the pivotal role of metabolite transfers. In many cases, perturbations of cytoplasmic transports are associated with the targeting of the *Cytoplasm-Nuclear Transport* module (Fig. 2). This result emphasizes again the major role metabolites play in signal transduction pathways. Nonetheless, as the *Cytoplasm-Nuclear Transport* module is never targeted on its own, it appears that the most critical reactions in these pathways occur within the cytoplasm.

Surprisingly, none of the MCS targeted the *IKK* module. The NF-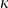
B and NF-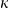
B(2) outputs can however be disabled by targeting this module, which activate the IKK complex. IKK is a protein kinase formed of three subunits that phosphorylates I
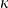
B, the inhibitory proteins of NF-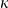
B [32]. Members of the I
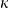
B family bind to NF-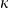
B to form an inactive complex. Two I
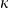
B proteins are present in the model: I
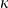
B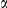 and I
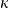
B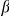, which correspond to the NF-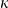
B and NF-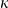
B(2) outputs, respectively. After phosphorylation by IKK, the NF-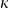
B/I
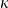
B complex rapidly dissociates, leaving NF-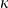
B free for activation. The phosphorylation of IKK occurs in the model through 11 independent reactions. However, the MCS algorithm failed to generate MCS with more than 8 reactions, hence being unable to produce some targeting *IKK*. One can argue that preventing the phosphorylation of IKK *in vivo* would require to shut down at least 11 different reactions each using a different enzymatic complex, which may not be an easy task. A simpler way to prevent IKK phosphorylation would be to disable the formation of the complex by knocking out one of its three subunits. This solution was not detected since the reaction leading to the formation of IKK is absent from the *ihs*TLR v1.0 model. Adding this reaction would have certainly provided us with more MCS for the NF-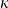
B outputs, thus increasing the number of EGS. Being able to generate MCS of higher cardinality represents an improvement that would greatly benefit the analysis of stoichiometric reconstructions. However, we would like to emphasize that MCS already give us access to information other techniques are unable to provide.

An additional limitation arises from the *ihs*TLR v1.0 model itself. This model is derived from the map of the TLR pathways developed by Oda and Kitano [10], [33]. However, this map does not exhaustively describe the pathways present in the immune system. Consequently, some crosstalk pathways, which may be affecting the producibility of the network's outputs, are potentially missing from the *ihs*TLR v1.0 model. Some EGS found from our computational results may prove to be irrelevant *in vivo* due to these missing components. Moreover, since the model is human centered, the mouse results must be interpreted with additional caution, although most of the TLR pathways are conserved between the human and mouse. Finally, a non-uniform gene nomenclature makes it difficult to exhaustively construct the GPR associations. We also want to emphasize that transient behaviors of the network do not affect the producibility of the outputs.

A valuable piece of information that we provide along with the EGS is the impact they have. Species, reaction, and module impacts were computed for every EGS. These numbers enable to choose gene sets whose knockout will have a minimal impact on the network. These EGS target a specific set of outputs while affecting the minimum number of species, reactions, and modules. We enumerated 801 EGS that targeted the power set of outputs with minimal impact, in both human and mouse (Dataset S2). Note that a minimal impact does not guarantee that the knockout will not be lethal for the cell. Linking the TLR network with a metabolic network could provide an answer to such a question. The integration of multiple systems is an efficient way to improve biological predictions of computational models [20], [34], [35].

Searching essential reactions or genes (*i.e.* MCS or EGS) for an objective has two applications. Firstly, this information can be used to disable the objective. Secondly, it can identify key elements to maintain in order to conserve the objective. Given the current literature, we can conjecture on the effect of disabling any of the outputs of the TLR network. NF-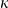
B is involved in a multitude of cellular processes: inflammation, T- and B-cells development, hematopoeitic cells survival, nitric oxide regulation, and lymphoid organogenesis [14]. Moreover, NF-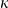
B is involved in the activation of other TFs present in the network. Hence, knocking out NF-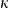
B may provoke serious repercussions on the host. In this case, the EGS identify deletions to avoid at all cost, or sets of genes that should not be targeted simultaneously in order to preserve the activity of NF-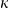
B. The knockout of AP-1 deregulates cell cycle and proliferation, and interferes with lymphoid cells development [11]. Here again, disabling AP-1 risks to provoke some unwanted consequences such as oncogenesis. Among all the outputs, ROS may be the one whose disabling causes the least side effects as it is the only one not being a TF. As a matter of fact, the oxidative burst generated by ROS provokes collateral damage to the host by itself [15]. Hence, blocking the formation of the NADPH oxidase complex would protect the organism from such damage, while depriving it of the positive effects of the burst.

We studied epistatic relations between modules of the *ihs*TLR v1.0 model, and determined knockout strategies to control the outcome of the TLR pathways. Analysis of the model suggests that the manipulation of the response of these pathways is best achieved by disabling targets downstream of the TLRs. We extended the Minimal Cut Sets to the notion of Essential Gene Sets (EGS), and determined lists of genes whose deletion perturbs combinations of the network's outputs. In addition, we assessed the impact of such deletions and provided the EGS that have minimal impact on adjacent pathways.

## Materials and Methods

### Signaling model

We represent a mass-balanced signaling network involving 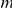 species and 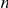 reactions with a stoichiometric matrix 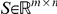. The stoichiometric matrix comprises the biochemical reactions occurring within the TLR pathways: receptor/ligand binding, phosphorylation cascades, activation of TFs. Each entry 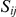 specifies the stoichiometric coefficient for species 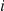 in reaction 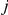. We represent the flux distribution through all the reactions by 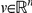, where the 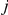-th component 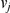 represents the flux through reaction 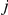. We consider all reactions to be irreversible (*i.e.* 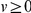) by breaking all reversible reactions into two irreversible ones. The concentration of each species in the system at time 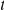 is given by 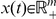. Under these assumptions, the change of concentration of species in time is given by
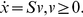
(1)Chemical reactions occurring in signaling networks (*e.g.* phosphorylation, transport, *etc*) take less than 1 sec, while transcriptional regulation and receptor internalization take on the order of 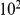 sec [18], [19]. This difference in time scales justifies the quasi-steady state assumption suggested by Li *et al.* [10], under which the fast signaling dynamics are assumed in steady state. This assumption is usually used in the study of metabolic networks, when the dynamics of the (fast) metabolic reactions are assumed in quasi-steady state when compared to the slow gene regulation dynamics [16]. Formally, under the steady state assumption, Equation (1) becomes:
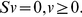
(2)All 14 inputs 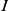 of the *ihs*TLR v1.0 model were considered *active*, *i.e.* 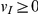, where 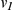 is the vector of all 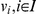. The inputs are 11 TLRs (TLR1 through TLR11), IL1R1, NOD1, and NOD2. Nine output reactions are defined in the model (Table 1). An output 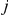 is considered *producible* if and only if
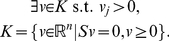
(3)The set 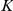 from Equation (3) is called the feasible flux cone of 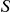.

### Minimal Cut Sets

Initially defined for metabolic networks, a cut set for an objective reaction is a set of reactions whose knockout disables that function [25]. Formally, 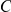 is a cut set for the objective reaction 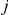 in model 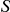 if and only if 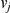 is producible in the wild type (*i.e.* 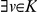 such that 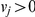) and
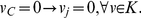
(4)A cut set is considered minimal if none of its subset is a cut set for that reaction (Fig. 1). MCS can be generated in a “brute force” way by performing all knockout combinations and by testing (*e.g.* using FBA) if the maximum flux through the objective reaction is zero. This method becomes rapidly infeasible for large networks and MCS of high cardinality. Alternatively, MCS can be constructed as minimal hitting sets of extreme pathways (EP) [25], which are the generators of the polyhedral cone from Equation (3). A hitting set for an objective reaction 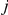 is a set that intersects all 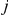-containing EP. This method has previously been employed to study epistasis in the *E. coli* and human metabolism [25]–[27]. We generated MCS following the algorithm detailed by Imielinski [26]. In summary, to compute MCS for a target reaction 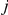, the algorithm performs three main steps: (1) generation of EP for an “over-approximated” cone 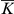 that includes the feasible flux cone 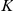, (2) computation of minimal hitting sets for reaction 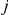 in 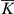, and (3) reduction of the above sets to minimality. As proved in [26], the sets obtained at (3) are guaranteed to be MCS for reaction 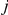 in 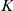. However, the approach is not complete, in the sense that it might miss some MCS.

### Essential Gene Sets

We constructed EGS iteratively for each MCS of all outputs. For each reaction in an MCS, we first identified combinations of genes whose deletion disabled that reaction. This was done by searching genes coding for proteins used in that reaction. We initially had to identify these genes in human and mouse through searches in the literature and in the Entrez Gene and MGI databases (Dataset S1). Similar sets of rules, usually called Gene-Protein-Reaction (GPR) associations, are present in many metabolic models [17], but were absent from the *ihs*TLR v1.0 model. Several combinations of genes may exist as different gene deletions can have the same effect over a reaction. We denote as 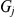 the set of all combinations for reaction 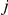. Once we obtained the sets 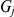 for all reactions in an MCS, we take their Cartesian product to construct EGS for the corresponding objective reaction. In the example from Figure 4, deleting A or B stops 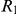 (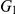 = 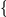
(A), (B)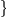) and deleting C stops 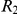 (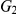 = 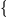
(C)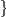). We obtain EGS for 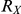 by taking the Cartesian product of 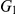 and 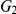: EGS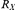 = 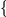
(A,C), (B,C)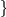. Note that the sets 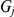 cannot be constructed for every reaction; at least one of the reaction substrates must be coded by a known gene. If at least one reaction cannot be disabled by gene deletion than no EGS can be generated. As MCS, EGS are considered to be minimal, *i.e.* no subset of an EGS is itself an EGS. We consider an EGS for a set of outputs as a set of genes whose deletion renders all the corresponding outputs non-producible.

### Estimation of gene deletions impact

In metabolic networks, the lethality of a gene deletion can be assessed by checking whether it disables the production of a biomass component. The *ihs*TLR v1.0 model does not include such an objective function. Moreover, this approach is relevant for the study of unicellular organisms, but for more complex organisms, the producibility of the biomass is only necessary, but not sufficient for survival. This motivated us to compute several estimates for the impact of the deletion of an EGS: the *species*, *reaction*, and *module* impacts (Dataset S2). We define these impacts as the number of species, reactions, and modules, respectively, that are no longer producible or affected upon deletion of all the genes in an EGS. The three estimates allow us to evaluate the global impact of gene deletions.

We determined which EGS have the “minimal” impact on the network based on the three impact values. Among all the EGS that knock out the same set of outputs, we first identified the ones that disabled either the minimum number of species (*i.e.* 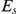), reaction (*i.e.* 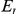), or module (*i.e.* 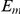). Then, taking the intersection 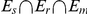 gave us the EGS with minimal impact. If this intersection is empty then no “minimal” EGS can be found.

## Supporting Information

Figure S1
**Classification of the MCS according to their cardinality and to the number of modules they target.** In each plot, the size of the dots shows the number of MCS containing 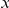 reactions that hit 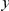 modules. 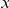 and 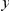 are given on the x- and y-axis, respectively. 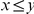 for all outputs since a reaction belongs to a single module. Having 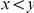 indicates that several reactions target the same module. The legend on the top-right corner provides with an estimate of the number of MCS according the size of the dot.(TIF)

Table S1 **Modules of the TLR network.** Function and description of the modules involved in the TLR network. Only modules hit by MCS are listed.
(PDF)

Table S2 **EGS validation.** List of the initial EGS obtained from our computation. Experimental validations of the predictions are found it the cited publications.
(PDF)

Dataset S1 **GPR associations of the TLR network.** List of the genes coding for the proteins of the TLR network. Entrez Gene ID are given for human and mouse.
(XLS)

Dataset S2 **EGS with minimal side effect.** List of the EGS that target the power set of outputs with minimal side effect in human (first sheet) and in mouse (second sheet). Each set of genes is followed by its respective species, reaction, and module impacts.
(XLS)
