# Supplementary figures and images for: Controlling the Outcome of the Toll-Like Receptor Signaling Pathways
Source: PLoS One. 2012 Feb 20;7(2):e31341. doi: 10.1371/journal.pone.0031341 (PMC3282698; doi:10.1371/journal.pone.0031341)

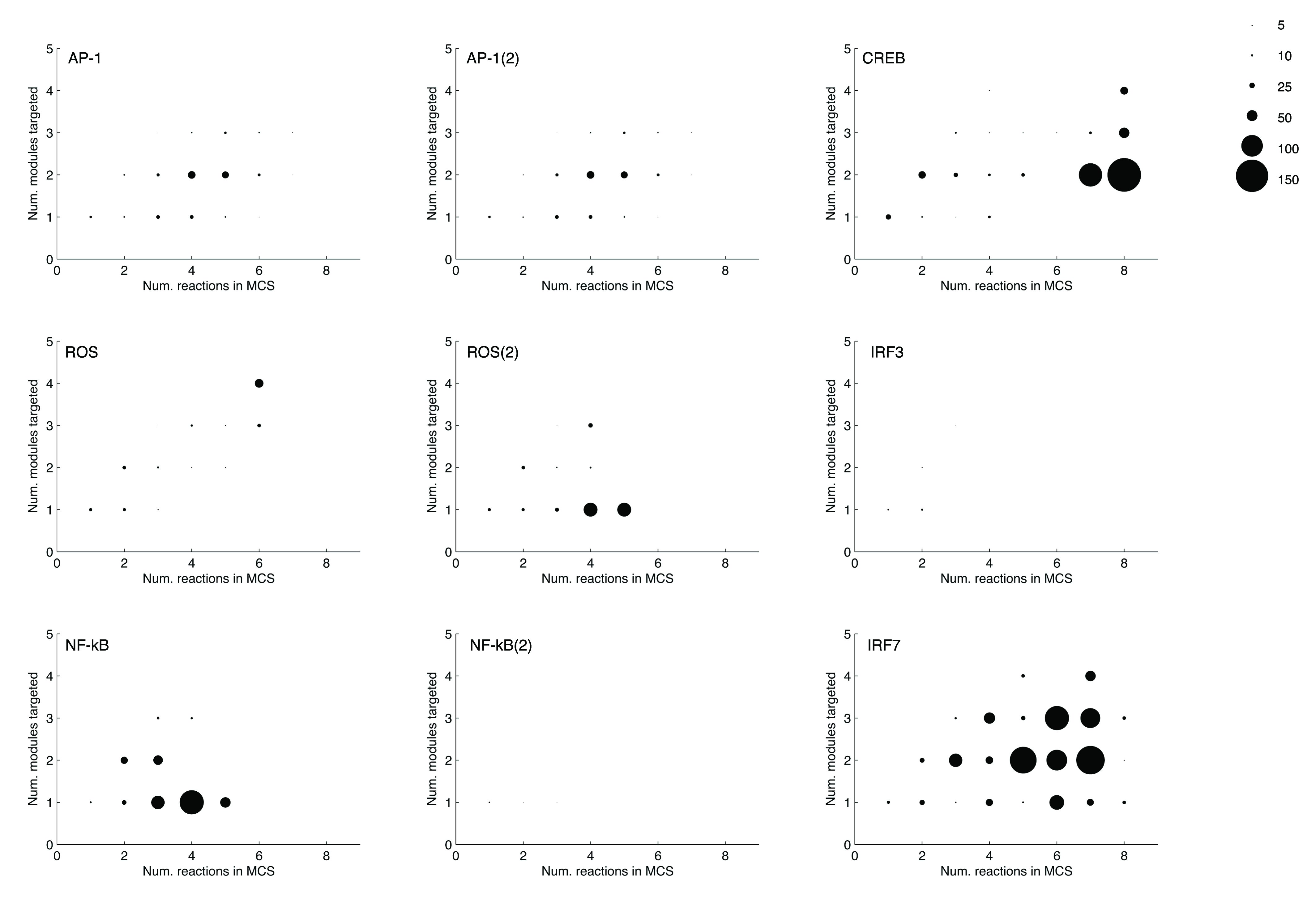

Supplement: Figure S1 — Classification of the MCS according to their cardinality and to the number of modules they target. In each plot, the size of the dots shows the number of MCS containing reactions that hit modules. and are given on the x- and y-axis, respectively. for all outputs since a reaction belongs to a single module. Having indicates that several reactions target the same module. The legend on the top-right corner provides with an estimate of the number of MCS according the size of the dot. (TIF) [file pone.0031341.s001.tif]
