# Supplementary material for: Controlling the Outcome of the Toll-Like Receptor Signaling Pathways
Source: PLoS One. 2012 Feb 20;7(2):e31341. doi: 10.1371/journal.pone.0031341 (PMC3282698; doi:10.1371/journal.pone.0031341)
Supplement: Table S1 — Modules of the TLR network. Function and description of the modules involved in the TLR network. Only modules hit by MCS are listed. (PDF) [file pone.0031341.s002.pdf]

Modules can be derived from the map of the TLR pathways: Figure S1 from Fan Li, Ines Thiele, Neema Jamshidi, and Bernhard O Palsson. Identification of potential pathway mediation targets in toll-like receptor signaling. PLoS Comput Biol, 5(2):e1000292, Feb 2009

**Module**	**Function**	**Description**
AP-1	Binding of AP-1 complex to AP-1 site	Activating Protein 1 (AP-1) is a family of transcription factors. AP-1 consist of heterodimers and homodimers of Jun, Fos, ATF, and JDP protein families [1, 2]. Homodimers are only formed by members of the Jun family. AP-1 complexes bind to AP-1 sites contained in promoters of various genes. AP-1 can be activated by cytokines [3], high glucose and angiotensin II [4], antioxidant conditions (stress signals) [5], or infection [6, 7].<br>The activation process generally occurs through the MAPKs pathways [8]. AP-1 has been shown to be associated with cell proliferation, differentiation, and inflammation [2, 4]. AP-1 is commonly described as a switch, deciding the fate of cells: survival or apoptosis [2, 9]. AP-1 can either induce apoptosis or promote cell proliferation depending on the cell type, the external stimuli, and the mixture of AP-1 dimers. Reactions in the modules include the phosphorylation of the Jun and Fos proteins by the MAPK pathways, the AP-1 complex formation, and binding to the AP-1 site.
Btk	Activation of Btk	Bruton's tyrosine kinase (Btk) is a tyrosine kinase involved in LPS signaling. Btk plays a role in the activation of NF-κB and AP-1, independently of MyD88, and is important for growth and differentiation of immune cells [10]. This module groups reactions necessary for the phosphorylation and ubiquitination of Btk
Calcium dependent cascade	Activation of calcium/calmodulin-dependent protein kinase	Calmodulins are calcium-binding proteins. The calcium/calmodulin complexes regulate calcium/calmodulin-dependent protein kinases, which are able to phosphorylate CREB [11, 12]. Reactions in this module include the binding of calcium to calmodulin and binding to calcium/calmodulin-dependent protein kinases.
Common metabolites	Transport of metabolites into the cytoplasm	Metabolites are necessary to model accurately the transduction of a signal [13]. This module allows the transport of metabolites across the cell membrane. Transport is achieve via channels or active transport.
CREB	Binding of CREB to CRE site	The cAMP response element binding protein (CREB) is a transcription factor which binds as a dimer to a specific response element called the cAMP response element (CRE). The best-known stimuli of CREB is cAMP which activate the catalytic subunit of the cAMP-dependent protein kinase (PKA). Once activated, PKA phosphorylates CREB in the nucleus which activates transcription of genes containing a CRE site [14]. Other stimuli can activate different kinases which all phosphorylate CREB. Among these stimuli are calcium, various growth factors, and survival and stress signals [15]. Although CREB is best known for its role in memory and complex behavior, it has been shown that CREB is involved in cell survival, proliferation, and cancer [15, 16, 17, 18]. Reactions in this module include dimerization and phosphorylation of CREB, and the binding of CREB and CBP to the CRE site.
Cytoplasm-Nucleus transport	Transport of various species into the nucleus	This module corresponds to the transport reactions allowing species from the network (metabolites and proteins) to cross the nuclear envelope. Transport is achieved via the nuclear pores.
Early endosome	Degradation and recycling of receptors	Endosomes are cellular compartment in eukaryotic cells where compounds are stored before degradation. They act as ``sorting stations'' where ligands are separated from their receptors. Receptors stay there until they are recycled or degraded [19].
GSK3β	Activation of GSK3β	Glycogen synthase kinase 3β (GSK3β) has been shown to play an important role for the phosphorylation of NF-κB [20]. Reactions in this module correspond to the phosphorylation (activation) of GSK3β and its transport between the cytoplasm and the nucleus.

IFN genes	Binding of IFN with the ISRE site	Interferons (IFN) are proteins expressed by lymphocytes and are involved in antiviral defense, cell growth, and immune activation. IFN are cytokines that enable cells to communicate with their neighbors and are described as the primary host defense mechanism in innate immunity [21]. IFN expression is controlled by a class of transcription factor known as the interferon regulatory factors (IRF). Nine IRF proteins have currently been identified, each having a specific role [22]. Reactions in this module include transport of the active form of IRF3 and IRF7 from the cytoplasm to the nucleus and their binding with their respective ISRE.
KSR1	Activation of the MAPK pathway	Kinase suppressor of Ras (KSR) is a conserved element involved in the Ras signaling pathway. It is use as a scaffold to facilitate activation of the MAPK pathway [23, 24]. Ras is a family of proteins sensible to growth factor stimuli and is involved in cell proliferation [25]. This module groups reactions of the Ras signaling pathway, including activation of the MAPK pathway.
Ligands	Import of ligands in the system	The ligands represent all the species that interact with the TLR. Ligands can be self-proteins and bacterial or viral particles. Reactions in this module include the import of ligands in the system.
Lipids	Lipids phosphorylation	Many proteins involved in signal transduction pathway can interact with lipids [10, 26, 27, 28]. This module groups reaction involved in the metabolism of lipids (mostly phosphorylation).
MAPK	Transmission of stimuli	Mitogen-Activated Protein Kinases (MAPKs) is family composed of three distinct subfamilies, each involved at a specific level. The MAPK pathways consist in cascades of phosphorylation events involving all three subfamilies. The MAPK kinase kinases (MKKKs) are first activated by specific stimuli and then phosphorylate MAPK kinases (MKKs). Once activated, MKKs phosphorylate MAKPs, which in turn activate various other proteins, including kinases, phospholipases, and transcription factors. Cells can respond to a variety of different stimuli by activating specific MAPK pathways [29]. Reactions in this module correspond to the various MAPK pathways and the phosphorylation cascade.
MAP3K7	Transmission of stimuli	MAP3K7 (also known as TAK1) is an ubiquitin-dependent kinase that can form a complex with TRAF6, TAB1 (also known as MAP3K7IP1), and TAB2 (also known as MAP3K7IP2). Once ubiquitinated, MAP3K7 can activate the IKK and JNK pathways [30]. This complex is thus linked to the activation of NF-κB and AP-1. This module contains reactions forming the MAP3K7/TRAF6/TAB1/TAB2 complex and its ubiquitination.
MyD88	Transmission of stimuli from the TLRs	MyD88 is a TIR domain-containing adaptor protein that is being recruited by the TLRs to transmit signals to specific kinases. MyD88 is involved in a wide range of signaling pathways, such as IL-1 [31], and is shown to be essential to induce a response to LPS in mice [32]. MyD88 also interacts directly with IRF7 to activate it [33]. This module groups all the reactions that are dependent from the recruitment of MyD88 by the TLRs.
NF-κB	Dissociation of NF-κB from IκB	The nuclear factor κB is a family of transcription factors involved in the immune and inflammatory responses. They consist in dimers formed by members the NF-κB family: RELA, NK-κB1, NF-κB2, c-REL, and RELB. NF-κB is in an inactive form when bound to one of the IκB proteins. These inhibitory proteins (IκBα, IκBβ, IκBε) retain the NF-κB complex in the cytoplasm [34]. NF-κB is activated in response to various stimuli (pathogens, stress signals, pro-inflammatory cytokines). A special family of kinases (IKK) is activated by these stimuli and phosphorylate IκB. IκB is ubiquitinated and then degraded as a result, leaving NF-κB free for phosphorylation (activation) and translocation to the nucleus. GSK3β, TBK1, IKK1, PKCζ proteins seems to play an important role in NF-κB phosphorylation [20]. NF-κB has a decisive role in the response of bacterial infections. It regulates the expression of many pro-inflammatory genes, such as cytokines, chemokines, or iNOS, and is important in the regulation of lymphocytes maturation [20]. NF-κB has also both pro- and anti-apoptotic effects and has been linked with cancer [35]. This module includes all the reactions related to NF-κB with the exception of the ones necessary for the phosphorylation of the complex, and the recruitment of IKK.

NF-κB phosphorylation	Activation of NF-κB	Once dissociated with its inhibitory protein IκB, NF-κB is free for phosphorylation and activation. This module groups the reactions necessary for the phosphorylation of NF-κB and its translocation to the nucleus.
PDK1	Activation of PDK1	Phosphoinositide-dependent protein kinase (PDK-1) is a serine/threonine kinase and a regulator of cell migration. It is linked to ROS [36] and has been shown to be activated by phospholipids [27]. Reactions in this module correspond to the activation by phospholipids and phosphorylation of PDK1.
PKCζ	Activation of PKCζ	Protein kinase C (PKC) is a family of enzymes which have an important role in several signal transduction pathways such as the Ras one [37]. The ζ isoform of this family (PKCζ) can be activated by various phospholipids and can influence gene expression in T-cells [28]. PKCζ is involved in the regulation of the MAPK pathway, and the NF-κB and AP-1 transcription factors. PKCζ has also been related to ROS production [38]. Reactions in this module include the activation (phosphorylation) of PKCζ by phospholipids and its transport between the cytoplasm and the nucleus.
Rho GTPases	Activation of GTPases	GTPases are a class of proteins which are in an active form when bound to GTP and in an inactive form when bound to GDP. Five family of GTPases exist in mammals: Ras, Rho, Rab, Arf, and Ran. This ``switch''-like behavior allows them to regulate many signal transduction pathways. They have been linked with the production of ROS and with the regulation of NF-κB and AP-1, thus regulating proliferation and cell survival pathways [39, 40]. Reactions in this module correspond to the cycles between active (GTP) and inactive (GDP) form of the Rho GTPases.
ROS production	Formation of the NADPH oxidase complex	Reactive oxygen species (ROS) are a class of highly reactive chemicals which contain oxygen. Most of them consist in superoxide anion, hydrogen peroxide, and hydroxyl radicals. ROS are a by-product of the mitochondrion activity. At high dose, ROS provoke oxidative damage which is harmful for the cells. At low dose they can regulate intracellular signal transduction pathways and act as second messenger molecules [41, 42]. Their production can be induced in immune cells by various stimuli leading to a ``respiratory burst''. This activity is used to kill pathogens, meanwhile provoking ``collateral damage''. ROS have also been described for their role in wound healing and platelets [26, 41].<br>ROS are synthesized by the NADPH oxidase complex (or phox complex) composed of various proteins: p22phox, gp91phox, p47phox, p67phox, and Rac2. The p47phox subunit is the site of many phosphorylation events which are necessary for NADPH oxidase activation [43, 44, 45]. This module groups reactions necessary for the formation of the phox complex. Most of the reactions correspond to the phosphorylation of p47phox.
Thioredoxin	Oxidation and reduction of thioredoxin	Thioredoxins are proteins with oxidoreductase activity and has been highly linked with ROS. They can have a growth factor-like effect and can stimulate the recruitment and proliferation of lymphocytes. Thioredoxins regulate AP-1, NF-κB, and the expression of some cytokines [46]. This module includes the necessary reactions for oxidation and reduction of thioredoxin.
TICAM	Transmission of stimuli from the TLRs and activation of IRF3	The TIR-containing adapter molecule (TICAM) is an adaptor molecule that transmits the signal from the TLRs to specific kinases. This pathway is independent from the MyD88 one. More specifically, TICAM-1 has been shown to transmit signals from TLR3 to IKKε and TBK1, both kinases responsible for the induction of IRF3 and NF-κB [47]. Reactions in this module comprise the transmission of the signal from TLR3 and TLR4 to IKKε and TBK1, as the phosphorylation of IRF3.
TLR	Binding of ligands with the TLRs	TLRs are a class of membrane proteins can bind to specific ligands, usually from viral or bacterial origins. Reactions in this module correspond to the binding of the TLRs with their corresponding ligands.

[1] M Karin, Z G Liu, and E Zandi. AP-1 function and regulation. Curr Opin Cell Biol, 9(2):240-6, Apr 1997

[2] Jochen Hess, Peter Angel, and Marina Schorpp-Kistner. Ap-1 subunits: quarrel and harmony among siblings. J Cell Sci, 117(Pt 25):5965-73, Dec 2004

[3] A von Knethen, D Callsen, and B Brune. NF-kappaB and AP-1 activation by nitric oxide attenuated apoptotic cell death in raw 264.7 macrophages. Mol Biol Cell, 10(2):361-72, Feb 1999

[4] J D Ahn, R Morishita, Y Kaneda, K U Lee, J Y Park, Y J Jeon, H S Song, and I K Lee. Transcription factor decoy for activator protein-1 (AP-1) inhibits high glucose- and angiotensin ii-induced type 1 plasminogen activator inhibitor (PAI-1) gene expression in cultured human vascular smooth muscle cells. Diabetologia, 44(6):713-20, Jun 2001

[5] Joseph Lunec, K Holloway, Marcus Cooke, and Mark Evans. Redoxregulation of DNA repair. Biofactors, 17(1-4):315-24, Jan 2003

[6] M. M Brinkmann, M Glenn, L Rainbow, A Kieser, C Henke-Gendo, and T. F Schulz. Activation of mitogen-activated protein kinase and nf- b pathways by a kaposi's sarcoma-associated herpesvirus k15 membrane protein. J Virol, 77(17):9346{9358, Sep 2003

[7] Jianping Xie, Hongyi Pan, Seungmin Yoo, and Shou-Jiang Gao. Kaposi's sarcoma-associated herpesvirus induction of AP-1 and interleukin 6 during primary infection mediated by multiple mitogen-activated protein kinase pathways. J Virol, 79(24):15027-37, Dec 2005.

[8] S Kim, S S Yu, I S Lee, S Ohno, J Yim, S Kim, and H S Kang. Human cytomegalovirus IE1 protein activates AP-1 through a cellular protein kinase(s). J Gen Virol, 80 (Pt 4):961-9, Apr 1999

[9] M Ameyar, M Wisniewska, and J B Weitzman. A role for AP-1 in apoptosis: the case for and against. Biochimie, 85(8):747-52, Aug 2003

[10] Caroline A Jefferies and Luke A J O'Neill. Bruton's tyrosine kinase (BTK)-the critical tyrosine kinase in lps signalling? Immunol Lett, 92(1-2):15-22, Mar 2004.

[11] M Sheng, M A Thompson, and M E Greenberg. CREB: a ca(2+)-regulated transcription factor phosphorylated by calmodulin-dependent kinases. Science, 252(5011):1427-30, Jun 1991.

[12] R P Matthews, C R Guthrie, L M Wailes, X Zhao, A R Means, and G S McKnight. Calcium/calmodulin-dependent protein kinase types II and IV differentially regulate CREB-dependent gene expression. Mol Cell Biol,14(9):6107-16, Sep 1994.

[13] Fan Li, Ines Thiele, Neema Jamshidi, and Bernhard O Palsson. Identification of potential pathway mediation targets in Toll-like Receptor signaling. PLoS Comput Biol, 5(2):e1000292, Feb 2009

[14] Marc Montminy and L M Bilezikjian. Binding of a nuclear protein to the cyclic-AMP response element of the somatostatin gene. Nature, 328(6126):175-8, Jan 1987

[15] B Mayr and Marc Montminy. Transcriptional regulation by the phosphorylation-dependent factor CREB. Nat Rev Mol Cell Biol, 2(8):599-609, Aug 2001

[16] Subhabrata Sanyal, David J Sandstrom, Charles A Hoeffer, and Mani Ramaswami. AP-1 functions upstream of CREB to control synaptic plasticity in drosophila. Nature, 416(6883):870-4, Apr 2002

[17] William A Carlezon, Ronald S Duman, and Eric J Nestler. The many faces of CREB. Trends Neurosci, 28(8):436-45, Aug 2005

[18] Michael D Conkright and Marc Montminy. CREB: the unindicted cancer co-conspirator. Trends Cell Biol, 15(9):457-9, Sep 2005

[19] James T Murray, Christina Panaretou, Harald Stenmark, Marta Miaczynska, and Jonathan M Backer. Role of Rab5 in the recruitment of hVps34/p150 to the early endosome. Traffic, 3(6):416-27, Jun 2002

[20] Qiutang Li and Inder M Verma. NF-kappaB regulation in the immune system. Nat Rev Immunol, 2(10):725-34, Oct 2002

[21] H Nguyen, J Hiscott, and P M Pitha. The growing family of interferon regulatory factors. Cytokine Growth Factor Rev, 8(4):293-312, Dec 1997

[22] A Paun and P M Pitha. The IRF family, revisited. Biochimie, 89(6-7):744-53, Jan 2007

[23] J Muller, S Ory, T Copeland, H Piwnica-Worms, and D K Morrison. C-TAK1 regulates Ras signaling by phosphorylating the MAPK scaffold, KSR1. Mol Cell, 8(5):983-93, Nov 2001

[24] Stephane Ory, Ming Zhou, Thomas P Conrads, Timothy D Veenstra, and Deborah K Morrison. Protein phosphatase 2A positively regulates Ras signaling by dephosphorylating KSR1 and Raf-1 on critical 14-3-3 binding sites. Curr Biol, 13(16):1356-64, Aug 2003

[25] D S Goodsell. The molecular perspective: the Ras oncogene. Oncologist, 4(3):263-4, Jan 1999

[26] Florian Krotz, Hae Young Sohn, Torsten Gloe, Stefan Zahler, Tobias Riexinger, Thomas M Schiele, Bernhard F Becker, Karl Theisen, Volker Klauss, and Ulrich Pohl. NAD(P)H oxidase-dependent platelet superoxide anion release increases platelet recruitment. Blood, 100(3):917-24, Aug 2002

[27] K E Anderson, J Coadwell, L R Stephens, and P T Hawkins. Translocation of PDK-1 to the plasma membrane is important in allowing PDK-1 to activate protein kinase B. Curr Biol, 8(12):684-91, Jun 1998.

[28] Bel en San-Antonio, Miguel A Iniguez, and Manuel Fresno. Protein kinase Czeta phosphorylates nuclear factor of activated T cells and regulates its transactivating activity. J Biol Chem, 277(30):27073-80, Jul 2002.

[29] Gary L Johnson and Razvan Lapadat. Mitogen-activated protein kinase pathways mediated by ERK, JNK, and p38 protein kinases. Science, 298(5600):1911-2, Dec 2002

[30] Chen Wang, L Deng, M Hong, G R Akkaraju, Jun-ichiro Inoue , Z J Chen. TAK1 is a ubiquitin-dependent kinase of MKK and IKK. Nature, 412(6844):346-51, 2001

[31] O Adachi, T Kawai, K Takeda, M Matsumoto, H Tsutsui, M Sakagami, K Nakanishi, and S Akira. Targeted disruption of the MyD88 gene results in loss of IL-1- and IL-18-mediated function. Immunity, 9(1):143-50, Jul 1998

[32] T Kawai, O Adachi, T Ogawa, K Takeda, and S Akira. Unresponsiveness of MyD88-defficient mice to endotoxin. Immunity, 11(1):115-22, Jul 1999

[33] Taro Kawai, Shintaro Sato, Ken J Ishii, Cevayir Coban, Hiroaki Hemmi, Masahiro Yamamoto, Kenta Terai, Michiyuki Matsuda, Jun ichiro Inoue, Satoshi Uematsu, Osamu Takeuchi, and Shizuo Akira. Interferon alpha induction through Toll-like Receptors involves a direct interaction of IRF7 with MyD88 and TRAF6. Nat Immunol, 5(10):1061-8, Oct 2004

[34] M D Jacobs and S C Harrison. Structure of an IkappaBalpha/NF-kappaB complex. Cell, 95(6):749-58, Dec 1998

[35] T D Gilmore. The Rel/NF-kappaB signal transduction pathway: introduction. Oncogene, 18(49):6842-4, Nov 1999

[36] David S Weber, Yoshihiro Taniyama, Petra Rocic, Puvi N Seshiah, Melissa A Dechert, William T Gerthoffer, and Kathy K Griendling. Phosphoinositide-dependent kinase 1 and p21-activated protein kinase mediate reactive oxygen species-dependent regulation of platelet derived growth factor-induced smooth muscle cell migration. Circ Res, 94(9):1219-26, May 2004

[37] P C Van Der Hoeven, J C Van Der Wal, P Ruurs, M C Van Dijk, and J Van Blitterswijk. 14-3-3 isotypes facilitate coupling of protein kinase C-zeta to Raf-1: negative regulation by 14-3-3 phosphorylation. Biochem J, 345 Pt 2:297-306, Jan 2000

[38] Janice Kwan, Hong Wang, Snezana Munk, Ling Xia, Howard J Goldberg, and Catharine I Whiteside. In high glucose protein kinase C-zeta activation is required for mesangial cell generation of reactive oxygen species. Kidney Int, 68(6):2526-41, Dec 2005

[39] Sandrine Etienne-Manneville and Alan Hall. Rho GTPases in cell biology. Nature, 420(6916):629-35, Dec 2002

[40] Saskia I J Ellenbroek and John G Collard. Rho GTPases: functions and association with cancer. Clin Exp Metastasis, 24(8):657-72, Jan 2007

[41] Chandan K Sen. The general case for redox control of wound repair. Wound Repair Regen, 11(6):431-8, Jan 2003

[42] T J Guzik, R Korbut, and T Adamek-Guzik. Nitric oxide and superoxide in inflammation and immune regulation. J Physiol Pharmacol, 54(4):469-87, Dec 2003

[43] J el Benna, L P Faust, and B M Babior. The phosphorylation of the respiratory burst oxidase component p47phox during neutrophil activation. J Biol Chem, 269(38):23431-6, Sep 1994

[44] L R Faust, J el Benna, B M Babior, and S J Chanock. The phosphorylation targets of p47phox, a subunit of the respiratory burst oxidase. J Clin Invest, 96(3):1499-505, Sep 1995

[45] S A Didichenko, B Tilton, B A Hemmings, K Ballmer-Hofer, and M Thelen. Constitutive activation of protein kinase b and phosphorylation of p47phox by a membrane-targeted phosphoinositide 3-kinase. Curr Biol, 6(10):1271-8, Oct 1996

[46] J Nordberg and E S Arner. Reactive oxygen species, antioxidants, and the mammalian thioredoxin system. Free Radic Biol Med, 31(11):1287-312, Dec 2001

[47] Katherine A Fitzgerald, Sarah M McWhirter, Kerrie L Faia, Daniel C Rowe, Eicke Latz, Douglas T Golenbock, Anthony J Coyle, Sha-Mei Liao, and Tom Maniatis. IKKepsilon and TBK1 are essential components of the IRF3 signaling pathway. Nat Immunol, 4(5):491-6, May 2003
