# Supplementary material for: Controlling the Outcome of the Toll-Like Receptor Signaling Pathways
Source: PLoS One. 2012 Feb 20;7(2):e31341. doi: 10.1371/journal.pone.0031341 (PMC3282698; doi:10.1371/journal.pone.0031341)
Supplement: Table S2 — EGS validation. List of the initial EGS obtained from our computation. Experimental validations of the predictions are found it the cited publications. (PDF) [file pone.0031341.s003.pdf]

EGS #	Genes in EGS	Outputs targeted	Validation	Comments
1	Fos	AP1	Gene is part of the output targeted	
2	Jun	AP1, AP1(2)	Gene is part of the outputs targeted	
3	MAPK8	AP1, AP1(2)	Dérijard B, Hibi M, Wub IH, Barrett T, Su B, Deng T, Karin M, Davis RJ (1994) **JNK1: A protein kinase stimulated by UV light and Ha-Ras that binds and phosphorylates the c-Jun activation domain**, Cell 76: 1025-1037	MAPK8 directly binds to Jun and activates it.
4	MAP2K4	AP1, AP1(2)	Lin A, Minden A, Martinetto H, Claret FX, Lange-Carter C, Mercurio F, Johnson GL, Karin M (1995) **Identification of a dual specificity kinase that activates the Jun kinases and p38-Mpk2**, Science 268: 286-90	MAP2K4 direclty activates MAPK8 (which activates Jun).
5	MAP2K7	AP1, AP1(2)	Tournier C, Whitmarsh AJ, Cavanagh J, Barrett T, Davis RJ (1997) **Mitogen-activated protein kinase kinase 7 is an activator of the c-Jun NH2-terminal kinase**, Proc Natl Acad Sci USA 94: 7337-42	MAP2K7 activates MAPK8 (which activates Jun).
6	IRAK1, TIFA, TRAF6, MAP3K7, TAB1, UBE2V1, TAB3, TAB2, UBE2N	AP1, AP1(2), IRF7		No validation found
7	CASP3, TRAF6, MAP3K3, MAP3K7, TAB1, UBE2V1, TAB3, TAB2, UBE2N	AP1, AP1(2), IRF7		No validation found
8	IRAK1, TRAF6, MAP3K3, MAP3K7, TAB1, UBE2V1, TAB3, TAB2, UBE2N	AP1, AP1(2), IRF7		No validation found
9	TRAF6, MAP3K1, MAP3K3, MAP3K7, TAB1, UBE2V1, TAB3, TAB2, UBE2N	AP1, AP1(2), IRF7		No validation found
10	TRAF6, MAP3K3, MAP3K7, ECSIT, TAB1, UBE2V1, TAB3, TAB2, UBE2N	AP1, AP1(2), IRF7		No validation found
11	ATF4	CREB	Gene is part of the output targeted	
12	CREB1	CREB	Gene is part of the output targeted	
13	CREB3	CREB	Gene is part of the output targeted	
14	CREB5	CREB	Gene is part of the output targeted	
15	IRF3	IRF3	Gene is part of the output targeted	
16	TBK1, IKKe	IRF3, IRF7	* Sharma S, tenOever BR, Grandvaux N, Zhou GP, Lin R, Hiscott J (2003) **Triggering the interferon antiviral response through an IKK-related pathway**, Science 300: 1148-51	RNAi experiment, simultaneous knockout of TBK1 and IKKe. Phosphorylation of IRF3 was inhibited and activity of IRF7 was decreased.
17	TICAM-1	IRF3, IRF7	Oshiumi H, Matsumoto M, Funami K, Akazawa T, Seya T (2003) **TICAM-1, an adaptor molecule that participates in Toll-like receptor 3-mediated interferon-beta induction**, Nat Immunol 4: 161-7	(partial validation) siRNA experiment, knockout of TICAM-1 decreases level of IFN-beta (IFN-beta promoters can be activated by IRF3).

18	TLR7,TLR8,TLR9	IRF7		No validation found
19	IRF7	IRF7	Gene is part of the output targeted	
20	TRAF6	IRF7	* Kawai T, Sato S, Ishii KJ, Coban C, Hemmi H, Yamamoto M, Terai K, Matsuda M, Inoue JI , Uematsu S, Takeuchi O, Akira S (20040 **Interferon-alpha induction through Toll-like receptors involves a direct interaction of IRF7 with MyD88 and TRAF6**, Nat Immunol 5:1061-8	Mutant experiment. Inactive mutants of TRAF6 inhibit activation of IRF7. Morever, TRAF directly interact with IRF7.
21	MyD88	IRF7	* Kawai T, Sato S, Ishii KJ, Coban C, Hemmi H, Yamamoto M, Terai K, Matsuda M, Inoue JI , Uematsu S, Takeuchi O, Akira S (2004) **Interferon-alpha induction through Toll-like receptors involves a direct interaction of IRF7 with MyD88 and TRAF6**, Nat Immunol 5:1061-8	Transfection experiment. IRF7 was activated only when transfected with MyD88. Moreover, MyD88 directly interact with IRF7.
22	IkBa	NF-kB		No validation found
23	beta-TrCP	NF-kB(2)	Wu C, Ghosh S (1999) **beta-TrCP mediates the signal-induced ubiquitination of IkappaBbeta**, J Biol Chem 274: 29591-4	Mutant experiment. Deletion mutants of beta-TrCP inhibits the activation of NF-kB.
24	IkBb	NF-kB(2)		No validation found
25	beta-TrCP, BTRC, UBE2D3	NF-kB, NF-kB(2)	* Wu C, Ghosh S (1999) **beta-TrCP mediates the signal-induced ubiquitination of IkappaBbeta**, J Biol Chem 274: 29591-4<br>* Nakayama K, Hatakeyama S, Maruyama SI, Kikuchi A, Onoé K, Good RA, Nakayama KI (2003) **Impaired degradation of inhibitory subunit of NF-kappa B (I kappa B) and beta-catenin as a result of targeted disruption of the beta-TrCP1 gene**, Proc Natl Acad Sci USA 100: 8752-7	(partial validation) Deletion mutants of beta-TrCP inhibits the activation of NF-kB (Wu and Ghosh). Reduced degradation of IkB(IkBalpha and IkBbeta) in BTRC(beta-TrCP1)-knockout mice (Nakayama *et al.*).
26	RELA	NF-kB, NF-kB(2)	Gene is part of the outputs targeted	
27	NFKB1	NF-kB, NF-kB(2)	Gene is part of the outputs targeted	
28	IKKa, IKKb, IKKg	NF-kB, NF-kB(2)		No validation found
29	PKCz	ROS	* Dang PMC, Fontayne A, Hakim J, El Benna J, Périanin A (2001) **Protein kinase C zeta phosphorylates a subset of selective sites of the NADPH oxidase component p47phox and participates in formyl peptide-mediated neutrophil respiratory burst**, J Immunol 166: 1206-13	Inhibition of PKCz reduced induced respiratory burst.
30	PKCa, PKCb, PKCd	ROS(2)		No validation found
31	PDK1	ROS(2)	Fujiyoshi M, Ozaki M (2011) **Molecular mechanisms of liver regeneration and protection for treatment of liver dysfunction and diseases**, J Hepatobiliary Pancreat Sci 18: 13-22<br>Kim JH, Chu SC, Gramlich JL, Pride YB, Babendreier E, Chauhan D, Salgia R, Podar K, Griffin JD, Sattler M (2005) **Activation of the PI3K/mTOR pathway by BCR-ABL contributes to increased production of reactive oxygen species**, Blood 105: 1717-23	Phosphorylation of mTOR is decreased in PDK1-knockout mice (Fujiyoshi and Ozaki). Inhibiton of mTOR reduces levels of ROS (Kim *et al.*).
32	NCF2	ROS, ROS(2)	Gene is part of the outputs targeted	

33	NCF4	ROS, ROS(2)	Gene is part of the outputs targeted	
34	CYBA	ROS, ROS(2)	Gene is part of the outputs targeted	
35	CYBB	ROS, ROS(2)	Gene is part of the outputs targeted	
36	RAC1	ROS, ROS(2)	Gene is part of the outputs targeted	
37	VAV1	ROS, ROS(2)	Kim C, Marchal CC, Penninger J, Dinauer MC (2003) **The hemopoietic Rho/Rac guanine nucleotide exchange factor Vav1 regulates N-formyl-methionyl-leucyl-phenylalanine-activated neutrophil functions**, J Immunol 171: 4425-30	Knockout experiment. Production of ROS is decreased in VAV1-knockout mice.
38	NCF1	ROS, ROS(2)	Gene is part of the outputs targeted	
39	BTK, SYKB	ROS, ROS(2)		EGS found only in mouse. No validation found
40	BTK, TEC	ROS, ROS(2)		No validation found
41	BTK, TXK	ROS, ROS(2)		No validation found
42	Src, SYKB	ROS, ROS(2)		EGS found only in mouse. No validation found
43	Src, TEC	ROS, ROS(2)		No validation found
44	Src, TXK	ROS, ROS(2)		No validation found

* These articles were also used for the reconstruction of the ihsTLR v1.0 model (Fan Li, Ines Thiele, Neema Jamshidi, and Bernhard O Palsson. Identification of potential pathway mediation targets in toll-like receptor signaling. PLoS Comput Biol, 5(2):e1000292, Feb 2009)
